# Experiences of Pharmacy Trainees from an Interprofessional Immersion Training

**DOI:** 10.3390/pharmacy6020037

**Published:** 2018-04-25

**Authors:** Daubney Boland, Traci White, Eve Adams

**Affiliations:** 1 Southern New Mexico Family Medicine Residency Program, Memorial Medical Center, Las Cruces, NM 88011, USA; daubney.harper@gmail.com; 2 College of Pharmacy, University of New Mexico, Albuquerque, NM 87106, USA; tmwhite@nmsu.edu; 3 Counseling & Educational Psychology Department, New Mexico State University, Las Cruces, NM 88003, USA

**Keywords:** interprofessional education, interprofessional collaboration, interprofessional care, communication, team-based training, teamwork, pharmacy

## Abstract

Interprofessional education is essential in that it helps healthcare disciplines better utilize each other and provide team-based collaboration that improves patient care. Many pharmacy training programs struggle to implement interprofessional education. This purpose of the study was to examine the effect of a 30-h interprofessional training that included pharmacy students to determine if the training helped these students build valuable knowledge and skills while working alongside other health care professions. The interprofessional training included graduate-level trainees from pharmacy, behavioral health, nursing, and family medicine programs where the trainees worked within teams to build interprofessional education competencies based on the Interprofessional Education Collaborative core competencies. Sixteen pharmacy trainees participated in the training and completed pre- and post-test measures. Data were collected over a two-year period with participants completing the Team Skills Scale and the Interprofessional Attitudes Scale. Paired sample *t*-tests indicated that, after this training, pharmacy trainees showed significant increases in feeling better able to work in healthcare teams and valuing interprofessional practice.

## 1. Introduction

Pharmacists play a critical role within the healthcare team in a variety of pharmacy practice settings. More importantly, their role on the interprofessional healthcare team has become increasingly influential in terms of ensuring patient safety and good health outcomes. Pharmacist interventions improve the process of patient care and clinical outcomes through the use of medication and therapeutic management, patient counseling, and professional health education [1]. Interactions between patients, healthcare professionals, other pharmacists and support personnel require effective communication skills to ensure patient safety and good health outcomes. Poor communication between physicians and pharmacists can lead to significant medication errors; therefore, a multidisciplinary approach has become essential in providing quality clinical care [2]. Due to the complexity of healthcare delivery and increasing prevalence of chronic diseases, interprofessional education (IPE) has been implemented in health professional education to provide the knowledge, skills, and attitudes to work effectively in a multidisciplinary setting [2,3]. Interprofessional education is defined by the World Health Organization (WHO) as when “students from two or more professions learn about from and with each other to enable effective collaboration and improve health outcomes” [4]. The WHO (2010) goes on to state that by learning how to work together interprofessionally, learners are better able to work in collaborative practice with other team members. 

As pharmacists are included as patient care providers on health care teams in order to evaluate complex drug regimens, it is important for the team to understand the role of the pharmacist in order to maximize the utilization of their clinical skills [5]. Team communication and integration of the pharmacist into ambulatory and hospital settings has been more successful than integrating community pharmacists into primary care; this may be due to having face-to-face time with providers within hospital settings, allowing pharmacists to communicate with other healthcare team members in person [6]. Additionally, there are often misperceptions about the role of the pharmacist, pharmacists being unclear about the roles of other healthcare professionals, lack of assertiveness by pharmacists and lack of team-based training [6]. By providing IPE during the training programs of healthcare professions, these misperceptions can lead to clarification of the impact the pharmacist can make on patient care as the medication expert on the interprofessional team. For the purpose of this article, we will discuss immersing pharmacy trainees into IPE activities with trainees of other healthcare disciplines likely to be encountered in professional practice and the impact this experience may have on their perceived skills to deliver patient-centered care utilizing a team-based approach. 

### Pharmacy within IPE

Pharmaceutical care is a philosophy of practice in which the patient is the primary beneficiary of the pharmacist’s actions which focuses the attitudes, behaviors, commitments, concerns, ethics, functions, knowledge, responsibilities and skills of the pharmacist on the provision of medication therapy. The primary goal of pharmaceutical care is achieving precise therapeutic outcomes toward patient health and quality of life [7]. Effective patient-centered interpersonal communication skills have been taught to pharmacists through a variety of IPE methods, including didactic learning modules, standardized patients, other experiential activities and didactic-based training. Standardized patient interaction is preferred over actual patients because the standardized patient is able to actively participate in teaching and assessment in real time [8]. The Accreditation Council for Pharmacy Education (ACPE) College of Pharmaceutical Education has recognized the importance of integrating IPE into the pharmacy curriculum stating “the need for interprofessional interaction is paramount to successful treatment of patients” but this training has not been consistently standardized or implemented in pharmacy education [9]. Furthermore, a workforce shortage within various healthcare fields has complicated the ability to integrate interprofessional care in daily practice [3]. 

The healthcare professions involved in IPE share in the same ideology of patient-centered care and therefore creates an ideal learning environment to work together, utilizing exemplary communications skills that allow the team to work together to focus on the patient’s needs [8]. Developing working relationships can be hindered by several factors including differing viewpoints on patient care, attitudes, and cultural beliefs between professions [2]. Pharmacists with experience working as part of a healthcare team have more favorable viewpoints on their role within the team structure and are less likely to see potential barriers to providing professional input due to other disciplines being overly protective of their own roles and thus not seeking that input from pharmacists [10].

## 2. Our Program—Interprofessional Immersion Training

The interprofessional immersion is a thirty-hour annual training developed by faculty from multiple programs including: The University of New Mexico College of Pharmacy, the Southern New Mexico Family Medicine Residency Program and several departments at New Mexico State University: The Counseling and Educational Psychology department, the School of Nursing department, the School of Social Work department, and the Anthropology department. Within these six departments, trainees are selected from each program to participate in the immersion: eight first-year family medicine residents beginning their residency, six third-year counseling psychology doctoral students beginning their primary care rotation, eight doctoral-level nurse practitioner students beginning clinical rotations, two social work students, seven pharmacy students and one clinical pharmacy resident, and five medical anthropology students who observed, facilitated focus groups, and collected qualitative data. 

This program began in 2013 and recently completed its fifth iteration. For the purpose of this article, we will report the findings on the 2016 and 2017 iterations as these are the years when we really refined the training to involve more team-based experiential activities and used the same outcome measures across both years. During both iterations of this training we had 32 trainees who were placed into eight groups of four, each with representation of pharmacy, nursing, behavioral health, and a family physician resident. Medical anthropology students observed, facilitated focus groups, and collected qualitative data. The aim of this training is to bring trainees who are about to begin clinical placement in medical settings together to learn important elements of interprofessional practice that can be translated to future work environments. Faculty trainers hoped that the immersion would increase the trainees team-based attitudes, expose them to team-based skills and provide some practice of these skills during the workshop that would help them work more effectively within their future healthcare teams.

The interprofessional immersion targeted all four core competencies identified by the Interprofessional Education Collaborative (IPEC) including: values and ethics, understanding roles and responsibilities, communication, and teamwork [11]. The educational components involve interactive didactics, insight-building, team-related activities, and simulated clinical practice that address each of these competencies. The training began with an activity that allowed trainees to discuss the roles of their professions and exercises that had them identify and discuss their professional values. Then they learned about communication tools such as SBAR (Situation, Background, Assessment, Recommendation) and CUS (Concerned, Uncomfortable, Safety issue), as well as specific components from Crucial Conversations and practiced communication role-plays [12]. We utilized team-building activities throughout the training, having each member work together within their team throughout each day. The trainees then participated in multiple patient simulations where they used individual clinical and IPE skills to assess and propose treatment with a standardized patient. Teams had the opportunity to practice working together while providing simulated patient care with live actors. 

Each iteration of the training had some minor variation in activities, but the core elements described above were included in the two years from which this data were collected. An Institutional Review Board approval for the research was obtained for both years and trainees completed pre and post measures, wrote in journals throughout the week, and participated in discipline-specific focus groups at the close of the training. The data presented in this article reflect the quantitative measures that were used as well as supplemental statements from students based on the qualitative data.

## 3. Method

### 3.1. Participants

Thirty-two trainees participate in this interprofessional immersion each year and this article focuses on the 16 pharmacy students who participated during the summers of 2016 and 2017. All trainees consented to participate in research. A demographics questionnaire was completed in addition to several questionnaires that were administered to the participants immediately before and after the immersion experience. The training was held at New Mexico State University (NMSU) in Las Cruces, New Mexico, which is located approximately 3 h south of the only college of pharmacy in New Mexico, the University of New Mexico (UNM) College of Pharmacy. Students were recruited to participate if they were completing an Introductory Pharmacy Practice Experience (IPPE) or Advanced Pharmacy Practice Experience (APPE) within the area or were taking the course for elective credit. This training is not mandatory for this pharmacy program, however, faculty and preceptors for the pharmacy program highly encourage their students to participate and they have been successful in recruitment of new students each year. 

For the 2016 cohort, the average age was 25, two identified as Asian, three as White/Non-Hispanic, and three as Hispanic. For the 2017 cohort the mean age was 25, with a range from 23 to 34. There were three male and five female, four participants identified as Hispanic, three reported being White/Non-Hispanic, and one identified as Biracial. Additionally, in the 2017 cohort, six of the eight pharmacy trainees reported planning to work in a primary care setting and within a rural community. 

### 3.2. Measures

*Team skills*. The Team Skills Scale (TSS) is a self-reporting measure used to assess participants’ self-assessment of their interprofessional team skills [13]. The measures consist of 17 items and uses a five-point Likert-scale, with (1) being poor and (5) being excellent. While it was originally developed to have three subscales: interprofessional skills, discipline-specific skills, and geriatric skills, it has since been used for its total score on team skills [14]. High scores on the TSS indicate that the participant reports a stronger ability to work within teams. Previous studies using the TSS total score found high internal consistency when completed by student and graduate health professionals (e.g., a Cronbach’s alpha of 0.95) [15]. For this study, the pre- and post-test alphas for the TSS were 0.82 and 0.91, respectively.

*Interprofessional Attitudes*. The Interprofessional Attitudes Scale (IPAS) is a self-report measure used to assess the attitudes of individuals as they relate to the interprofessional education competencies [16]. The IPAS is a 27 item instrument that uses a five-point Likert scale ranging from (1) strongly disagree to (5) strongly agree. There are five subscales: (1) Teamwork, Roles and Responsibilities; (2) Patient-Centeredness; (3) Interprofessional Biases; (4) Diversity and Ethics; and (5) Community-Centeredness. These attitudes are only measured at the subscale level so there is no total score. Higher scores on each subscale indicate a greater espousal of the attitude related to that particular domain of interprofessionalism. Because the focus of this study’s interprofessional immersion did not include community-based healthcare or diversity in healthcare, we did not include these subscales in our hypotheses. Additionally, we were only interested in one item of the Interprofessional Biases subscale (Item #15, “Health professionals/students from other disciplines have prejudices or make assumptions about me because of the discipline I am studying”), so we did not use the entire Biases subscale. In the instrument development study of the IPAS, the internal consistency alphas for the subscales ranged from 0.62 to 0.92. For this study, the pre- and post-test alphas for the Teamwork subscale were 0.88 and 0.70, respectively and on the Patient Centeredness subscale they were 0.90 and 0.96.

*Supplemental Data.* In addition to quantitative data, qualitative data were collected in the form of journals. Each student kept a journal throughout the training and responded to specific questions pertaining to IPE such as, “what was the most surprising thing you learned about another profession today; what stands out to you most about your experience with patient simulations today in terms of working with your team; compare your experience with patient simulations from yesterday and today.” Due to the limited scope of this article, qualitative statements from students will be used as a supplement to the quantitative findings. 

### 3.3. Hypotheses and Data Analysis

Using the statistical software program SPSS, paired sample *t*-tests were conducted to analyze the quantitative data collected in the form of the two self-report measures. Our hypotheses were that there would be a significant increase from pre-test to post-test in participants’ self-reported team skills on the TSS and in positive attitudes on the teamwork, roles, and responsibilities subscale of the IPAS. In order to assess for a general placebo effect, where participants would respond more favorably on all items at the time of post-test; we hypothesized that there would be no change on the Patient-Centeredness subscale of the IPAS because the immersion focus was on team-skills. There should be no reason for Patient-Centeredness to increase, except due to a general placebo effect. While we could have utilized the entire Biases subscale, we were particularly interested in item #15 of this IPAS subscale, which states, “Health professionals/students from other disciplines have prejudices or make assumptions about me because of the discipline I am studying.” If the immersion was successful in creating an appreciation of all the healthcare team professions, then we hypothesized that there would be no increase on this item. In other words, a successful interprofessional immersion minimally should not increase any professional biases so as to make trainees feel more alienated or judged by the trainees in other professions after having interacted with each other during the immersion.

From a qualitative viewpoint, reflections in student journals were reviewed to evaluate how the pharmacy students identified with their role on the team and their perceived abilities to participate in future interprofessional collaborations due to a better understanding of team-based care.

## 4. Results

A significant difference was found in pharmacy trainees’ reported team skills [t (15) = −7.26, *p* = 0.001] as evidenced by an increase in TSS scores from pre-test (M = 3.51, SD = 0.38) to post-test (M = 4.30, SD = 0.43). Similarly, there was a significant increase in the pharmacy trainees reported attitudes indicating that they valued working within a healthcare team [t (15) = −3.18, *p* = 0.006] with the pre-test IPAS teamwork scores being lower (M = 4.41, SD = 0.45, than the post-test scores (M = 4.75, SD = 0.29). See Table 1 for the *t*-test results.

Regarding our exploration of the null hypothesis that there would be no significant differences between pre- and post-test scores on the patient-centeredness subscale of the IPAS and Item #15 of the Bias subscale of the IPAS (i.e., perceptions of other professions being biased about one’s own profession), the null hypothesis was supported. There was no significant difference on the participants’ attitudes about being patient-centered [t (15) = 0.09, *p* = 0.933], with pre-test scores (M = 4.76, SD = 0.37) being virtually the same as the post-test scores (M = 4.75, SD = 0.46). Similarly, there was no significant difference on Item 15 [t (15) = 0.00, *p* = 1.00] with pre-test scores regarding perceptions of professional bias (M = 3.38, SD = 1.15) being exactly the same as the post-test scores (M = 3.38, SD = 1.15).

In regards to the journals, there were comments that reflected changes in the perception of working within healthcare teams. Practicing together helped reduce hierarchy often experienced in healthcare. One pharmacy student reflected,

“During this week hierarchy was not an issue. My interprofessional team was very mindful of the importance of each team members’ contributions. Having this mindset enabled for trust and positive relations to form from the beginning. From my experience outside of this week, healthcare is very ‘hierarchical,’ which complicates patient care.”

Another pharmacy student reflected,

“Everyone was able to step back and accommodate to the needs of the team we all worked very well together and were able to see our strengths and weaknesses and build on them to improve teamwork.”

Pharmacy trainees also reflected on how their perceptions of working within teams had changed as a result of this training. In regards to an ability to work as a team, one of the pharmacy trainees reflected,

“I think fully understanding what each discipline is an expert on and their role and then seeing them as tools rather than a challenge to your patient care process will have a very positive impact on patient care. It is going to be very interesting to see the future outcomes of health professionals who are trained as a team from the beginning of their education.”

Another student reflected on how working within a team was both surprising and helped her be better in her own role. The pharmacy trainee stated,

“Working with my team in today’s simulation had the opposite effect of what I was expecting. I had previously thought teamwork and being put on the spot in front of other healthcare providers would be more nerve racking than an individual patient encounter, but surprisingly it was less stressful and the combined brain power proved to provide better, more encompassing care and was actually enjoyable for me.”

This pharmacy student reflected on how the experience helped him feel more comfortable in his role within the team and affirmed that team-based care can be an effective way to practice. He stated,

“I felt that I fit very well in my role as a pharmacy student with my team. I think that I complement their focuses very well and make a great impact with medication and lifestyle management. They asked a lot of good questions that helped me think more about my assessment. I also think we all did not overstep boundaries and didn’t try to talk over one another and didn’t belittle one another either. I feel that I appreciate the roles of the team members [much] more after today.”

Lastly, a family medicine physician resident made this journal reflection regarding the importance of knowing one’s team members and their roles as well as the importance of having a shared goal.

“Working in a team involves many ‘moving parts.’ It would never work unless each team member buys into the team and the team shares a common goal. My team has bonded really well and has learned a lot about each other. This helped us greatly during the simulation. It is not enough to just know your role in the team, but you must also know others’ roles as well.”

## 5. Conclusions

Results on the Team Skills Scale (TSS), the Interprofessional Attitudes Scale (IPAS), and comments in their journals highlight that this interprofessional training produced some positive change in pharmacy trainees’ perceptions about their ability and desire to work on interprofessional healthcare teams. The results provide empirical support that even a relatively brief interprofessional training can produce changes in pharmacy students’ perspectives in working within healthcare teams, both in terms of their self-efficacy regarding their team-based skills and in their attitudes about the importance of team-based care. This is consistent with previous studies utilizing the Team Skills Scale (TSS) and qualitative responses to interprofessional training with pharmacy professionals [14]. Robben et al. reported significantly higher perceptions of team skills after their interprofessional training, as well as students described increased awareness/appreciation of the lenses and functions of other professions.

While there were a small number of participants, the differences in pre- and post-test scores were significant enough to be detected. In particular, the average score for participants’ self-report of their team skills on the TSS at pre-test was mid-way between “Good” and “Very Good”, whereas the average post-test score was mid-way between “Very Good” and “Excellent.” Regarding attitudes about teamwork on the IPAS the participants were closer to “Agree” at the pre-test and then closer to “Strongly Agree” at post-test. While there was no control group, it is important to note that other subscales (i.e., patient-centeredness) did not change from pre- to post-test. In a controlled trial evaluation where pre-licensed health service students received either 11 hours of interprofessional training or their discipline’s normal curriculum, Darlow et al. reported significantly heightened mean differences on the TSS in the intervention group versus the control group [17]. The authors feel that this may be due to the fact that the trainees already possessed strong values and attributes of patient-centeredness. This could also be due to the fact that pharmaceutical care aims to be patient-centered and this is already delivered throughout the pharmacy curriculum. The authors hypothesized that there would be not significant differences from pre- to post-test in this particular area. This support of the null hypothesis highlights the importance of tying interprofessional skill improvement to improved patient care when engaging pharmacy trainees in IPE. Some research points to mixed findings around changes in health service students’ attitudes in response to shorter interprofessional trainings (e.g., three-day training vs. one-year), with some medical students reporting no attitude change towards interprofessional teamwork in shorter trainings and heighted positive attitudes in longer trainings [18,19]. However, in this study it is more likely that pharmacy students’ patient centeredness remained constant, regardless of length of training, evidenced by journal entries highlighting how students felt they could work more effectively and comfortably within healthcare teams.

Pharmacy journal entries highlighted how students felt they could work more effectively and comfortably within healthcare teams and how doing so would improve the quality of the healthcare for patients. This qualitative theme complements our TSS and IPAS results, in which pharmacy trainees reported increased team skills and increased attitudinal valuation of working on an interprofessional healthcare team. A study utilizing the IPAS has reported that students’ perceptions of overall interprofessional competence increases significantly after IPE trainings, and that as perceptions of competence increased, attitudes toward teamwork also become more positive [20].

Additionally, we were specifically interested in assessing if the immersion training had a negative effect on the pharmacy trainees’ perceptions of how pharmacy was viewed by trainees in other healthcare professions. The results of paired sample *t*-test on Item 15 of the IPAS indicated that pharmacy trainees neither agreed nor disagreed with this statement at both pre- and post-test. Thus, this brief immersion did not create more negative biases about the other healthcare professions. Ideally, participants should ultimately disagree with this statement, feeling that the trainees of other professions have positive perceptions of their own discipline. This finding suggests that when planning interprofessional immersion trainings the faculty should directly address any biases in debriefings with trainees to determine if there might be specific activities that would help address this concern. For example, at the end of an immersion it might be important to have trainees from each of the healthcare professions sharing specifically positive perceptions they now have about other professions as a result of interacting in the team-based activities. 

A recent study by Peeters et al. suggests that delivering interprofessional education focused in smaller groups within a larger classroom-based course was helpful for students to achieve learning objectives based on IPEC competencies and student satisfaction with the course [21]. Our study adds to the IPE literature because it demonstrates the positive impact of a briefer training IPE experience, delivered in small group, team-based activities with four healthcare disciplines. More research is needed to determine what active ingredients of IPE trainings are most effective in creating the increases in self-efficacy related to team skills, as well as more positive attitudes about team-based work. For example, in our interprofessional immersion, we provided many real-world examples of team-based care as the faculty trainers work in teams in their daily professional practices and model these relationships and communication skills with fellow faculty throughout the course. Future research could leave this component out of the interprofessional immersion to see if this negatively impacted the change in scores from pre- to post-test.

A limitation of the current study is that the outcomes measures did not directly assess changes in communication styles or habits, nor the trainees’ ability to work in his or her specific work-setting. The trainers hope that the skills learned will transfer to other work settings and that through practice these skills will be more comfortable and likely to be utilized. Pecukonis argues that it can be difficult to translate interprofessional education into practice [22]. By providing this training at the start of the trainees’ clinical experiences in interprofessional settings, we hope they will be able to apply these team skills immediately in their clinical setting. To gather empirical support for this goal, future research needs to incorporate an assessment of the participants’ team-based skills as well as gather longitudinal data from participants. In order to enhance our understanding of the specific team-based skills learned during our training, our faculty hopes to analyze data collected from video recordings of trainees working together during patient simulations as well as collect data from past graduates that participated in this interprofessional training. There is little research on IPE that assesses actual skills observed by independent raters, particularly in “real-world” settings.

The trainees in our study varied in their years of pharmacy education, ranging from completing their first year of pharmacy school to beginning practice as a pharmacy resident. This variation may have affected participants’ scores on the TSS and IPAS due to variance in knowledge, skills and/or confidence levels. Although the training emphasized a focus on team-based skills and communication rather than professional knowledge-based skills, these differences in training level may impact participants’ self-reporting of team-based skills and attitudes. With larger sample sizes, it will be possible to examine the impact of participants’ training level. The research on IPE has not determined when is the optimal time for such training to occur.

There is great importance in building interprofessional education skills to help reduce medical errors, increase communication, provide better patient care and reduce provider burn-out. One way to enhance these skills is through specific training surrounding the interprofessional education competencies. This study provides evidence that brief, team-based learning can be an effective modality for learning these skills, building trainees’ confidence in being able to work within healthcare teams, and creating a greater appreciation for this type of healthcare delivery.

## Figures and Tables

**Table 1:** Paired-sample *t*-test results for interprofessional immersion variables.

	Before Immersion Pre-Test (*n* = 16)		After Immersion Post-Test (*n* = 16)		
Variable	M	SD	M	SD	t
Team Skills Scale	3.51	0.38	4.30	0.43	−7.26 ***
IPAS Teamwork	4.41	0.45	4.75	0.29	−3.18 **
IPAS Patient-Centered	4.76	0.37	4.75	0.46	0.09
IPAS Item #15	3.38	1.15	3.38	1.15	0.00

Note: TSS = Team Skills Scale (potential range of scores 1–5); IPAS = Interprofessional Attitudes Scale (potential range of scores 1–5); Item 15: “Health professionals/students from other disciplines have prejudices or make assumptions about me because of the discipline I am studying” (potential range of scores 1–5); ** *p* < 0.01, *** *p* < 0.001.

## References

[1] Nikansah N., Mostovetsky O., Yu C., Chheng T., Beney J., Bond C.M., Bero L. (2010). Effect of outpatient pharmacists’ non-dispensing roles on patient outcomes and prescribing patterns. Cochrane Database Syst. Rev..

[2] Gallagher R.M., Gallagher H.C. (2012). Improving the working relationship between doctors and pharmacists: Is inter-professional education the answer?. Adv. Health Sci. Educ..

[3] Page R.L., Hume A.L., Trujillo J.M., Leader W.G., Vardeny O., Neuhauser M.M., Dang D., Nesbit S., Cohen L.J. (2009). Interprofessional education: Principles and application a framework for clinical pharmacy. Pharmacother. J. Hum. Pharmacol. Drug Ther..

[4] World Health Organization (2010). Framework for Action on Interprofessional Education and Collaborative Practice.

[5] Farrell B., Ward N., Dore N., Russell G., Geneau R., Evans S. (2013). Working in interprofessional primary health care teams: what do pharmacists do?. Res. Soc. Adm. Pharm..

[6] Jorgenson D., Dalton D., Farrell B., Tsuyuki R., Dolovich L. (2013). Guidelines for pharmacists integrating into primary care teams. Can. Pharm. J..

[7] Hepler C.D., Strand L.M. (1990). Opportunities and responsibilities in pharmaceutical care. Am. J. Health-Syst. Pharm..

[8] Hess R., Hagemeier N.E., Blackwelder R., Rose D., Ansari N., Branham T. (2016). Teaching communication skills to medical and pharmacy students through a blended learning course. Am. J. Pharm. Educ..

[9] Accreditation Council for Pharmacy Education (2015). Accreditation Standards and Key Elements for the Professional Program in Pharmacy Leading to the Doctor of Pharmacy Degree “Standards 2016”. https://www.acpe-accredit.org/pdf/Standards2016FINAL.pdf.

[10] Dobson R., Henry C., Taylor J., Zello G., Lachaine J., Forbes D., Keegan D. (2006). Interprofessional health care teams: Attitudes and environmental factors associated with participation by community pharmacists. J. Interprof. Care.

[11] Interprofessional Education Collaborative (IPEC) Expert Panel (2011). Core Competencies for Interprofessional Collaborative Practice: Report of an Expert Panel.

[12] Patterson K. (2012). Crucial Conversations: Tools for Talking When Stakes Are High.

[13] Hyer K., Heinemann G.D., Fulmer T., Heinemann G.D., Zeiss A.M. (2002). Team Skills Scale. Team Performance in Health Care: Assessment and Development.

[14] Robben S., Perry M., van Nieuwenhuijzen L., van Achterberg T., Rikkert M.O., Schers H., Heinen M., Melis R. (2012). Impact of interprofessional education on collaboration attitudes, skills, and behavior among primary care professionals. J. Contin. Educ. Health.

[15] Fulmer T., Hyer K., Flaherty E., Mezey M., Whitelaw N., Jacobs M.O., Luchi R., Hansen J.C., Evans D.A., Cassel C. (2005). Geriatric interdisciplinary team training program evaluation results. J. Aging Health.

[16] Norris J., Carpenter J.G., Eaton J., Guo J.W., Lassche M., Pelt M.A., Blumenthal D.K. (2015). The development and validation of the interprofessional attitudes scale: Assessing the interprofessional attitudes of students in the health professions. Acad. Med. J. Assoc. Am. Med. Coll..

[17] Darlow B., Coleman K., McKinlay E., Donovan S., Beckingsale L., Gray B., Neser H., Perry M., Stanley J., Pullon S. (2015). The positive impact of interprofessional education: A controlled trial to evaluate a programme for health professional students. BMC Med. Educ..

[18] Park J., Hawkins M., Hamlin E., Hawkins W., Bamdas J. (2014). Developing positive attitudes toward interprofessional collaboration among students in the health care professions. Educ. Gerontol..

[19] Shrader S., Griggs C. (2014). Multiple interprofessional education activities delivered longitudinally within a required clinical assessment course. Am. J. Pharm. Educ..

[20] Coiro M.J., Preis J. (2018). Increases in graduate students’ interprofessional competence associated with clinical training activities. Health Interprof. Pract..

[21] Peeters M.J., Sexton M., Metz A.E., Hasbrouck C.S. (2017). A team-based interprofessional education course for first-year health professions students. Curr. Pharm. Teach. Learn..

[22] Pecukonis E. (2014). Interprofessional education: A theoretical orientation incorporating professional-centrism and social identity theory. J. Law Med. Ethics.

